# Chloroplast genome of silverleaf nightshade (*Solanum elaeagnifolium* Cav.), a Weed of National Significance in Australia

**DOI:** 10.1080/23802359.2020.1775527

**Published:** 2020-06-17

**Authors:** Xiaocheng Zhu, Aisuo Wang, Hanwen Wu, Jianmin Lin

**Affiliations:** a Graham Centre for Agricultural Innovation (Charles Sturt University and NSW Department of Primary Industries), Charles Sturt University, Wagga Wagga, Australia; b NSW Department of Primary Industries, Wagga Wagga Agricultural Institute, Wagga Wagga, Australia; c College of Mathematical Science, Hua Qiao University, Quanzhou, Fujian, P.R. China

**Keywords:** Chloroplast genome, silverleaf nightshade, *Solanum elaeagnifolium*, *Leptostemonum*

## Abstract

*Solanum elaeagnifolium* Cav. is a widely distributed weed and recognized as a Weed of National Significance in Australia. This study sequenced the chloroplast (*cp*) genome of *S. elaeagnifolium,* which is 155,049 bp in length, including a large single-copy region at 85, 426 bp, a small single-copy region at 18,419 bp and two inverted repeats at 25,602 bp. A total of 130 genes were annotated. The phylogeny among the *S. elaeagnifolium* and 42 *Solanum* chloroplast genomes suggested *S. elaeagnifolium* is closely related to *Solanum* species from the section of Melongena.

Silverleaf nightshade (*Solanum elaeagnifolium* Cav.) is one of the worst agricultural weeds around the world. It is native to North America and is widely distributed beyond its native range (Gopurenko et al. 2014). In Australia, *S. elaeagnifolium* is listed as a Weed of National Significance and causes up to 77% yield loss in cereals (Stanton et al. 2009). The lack of a reliable method to distinguish *S. elaeagnifolium* from other *Solanum* species, however, often resulted in misidentification (X Zhu et al. 2018, XC Zhu et al. 2011) and caused unnecessary delay in control and missed the prime opportunity of early eradication (Hosking et al. 1996).

Chloroplast (*cp*) genome is a circular DNA molecular that generally ranged from 120 to 160 kbp, typically consisting of a large and a small single-copy regions (LSC and SSC), and two inverted repeats (IRs). *cp* genome is highly conserved and lacks recombination. Therefore, it has been used to develop DNA barcodes for the identification purpose (Raubeson and Jansen 2005). More completed *cp* genomes are available due to the reduced time and cost of the next-generation sequencing technology.

In our study, we obtained the complete *cp* genome sequence of *S. elaeagnifolium*. This study provides information for the future development of molecular tools to improve the identification, thereby contributing to the effective management of *S. elaeagnifolium*.

Fresh leaf sample of *S. elaeagnifolium* was collected from Wagga Wagga, New South Wales, Australia (N –35.101776, E 147.386991). This sample was preserved at Wagga Wagga Agricultural Institute (voucher ww19479). Genomic DNA was extracted using a modified CTAB method (Doyle 1987). Sequencing of the genomic DNA was performed using an Illumina Hiseq2000 platform at Beijing Genomics Institute (BGI, Hong Kong). A total of 13,324,346 raw reads were generated with an average read length of 125 bp. The raw reads were subjected to the quality-control process by readfq v5 (https://github.com/lh3/readfq) to trim adapter sequence, duplications and low-quality read. The *cp* genome was obtained by *de novo* assembly using SOAPdenovo2 with k-mer size optimized to 61 (Luo et al. 2012). The assembled *S. elaeagnifolium cp* genome was 155,049 bp in length, consisting of a 85,426 bp LSC, a 18,419 bp SSC regions and two IRs at 25,602 bp. The GC content of *S. elaeagnifolium cp* genome was 37.8%, with the highest GC content found in the IR region (43.1%), followed by LSC at 35.8% and SSC at 32.0%. The assembled chloroplast genome was deposited into GenBank under the accession number of KX792501.

The *S. elaeagnifolium cp* genome was annotated with CpGAVAS using *S. bulbocastanum*, *S. lycopersicum* and *S. tuberosum* as references with default settings (Liu et al. 2012), followed by verification using local BLAST and manual adjustments. A total of 108 distinct genes were annotated, including 4 ribosomal RNA genes, 28 transfer RNA genes, and 78 protein-coding genes. Duplicated genes in the *cp* genome include nine protein-coding genes, seven tRNA genes, and four rRNA genes, making a total of 130 genes. Introns were found in nine protein-coding genes *atpF*, *rpoC1*, *ycf*3, *clpP*, *rpl*2, *ndhA* and *ndhB*.

Sequences of *S. elaeagnifolium* and 42 published *Solanum* species were aligned using MAFFT v7 (Katoh and Standley 2013) and phylogeny were inferred using MrBayes v3.2 using GTR + I + G model (Ronquist and Huelsenbeck 2003). *Capsicum annuum* (MH559323) was used as an outgroup. Indels were excluded for analysis. Four clades were formed in the phylogenetic trees (Figure 1). *Solanum tuberosum, S. lycopersicum* and related species (*Potato* major clade) were clustered into separated clades. *Solanum nigrum* (*Morelloid* major clade) and *S. dulcamara* (*Dulcamaroid* major clade) were grouped together. *Solanum elaeagnifolium* and *S. melongena* related species from the major clade of *Leptostemonum* formed a highly supported clade.

**Figure 1.**
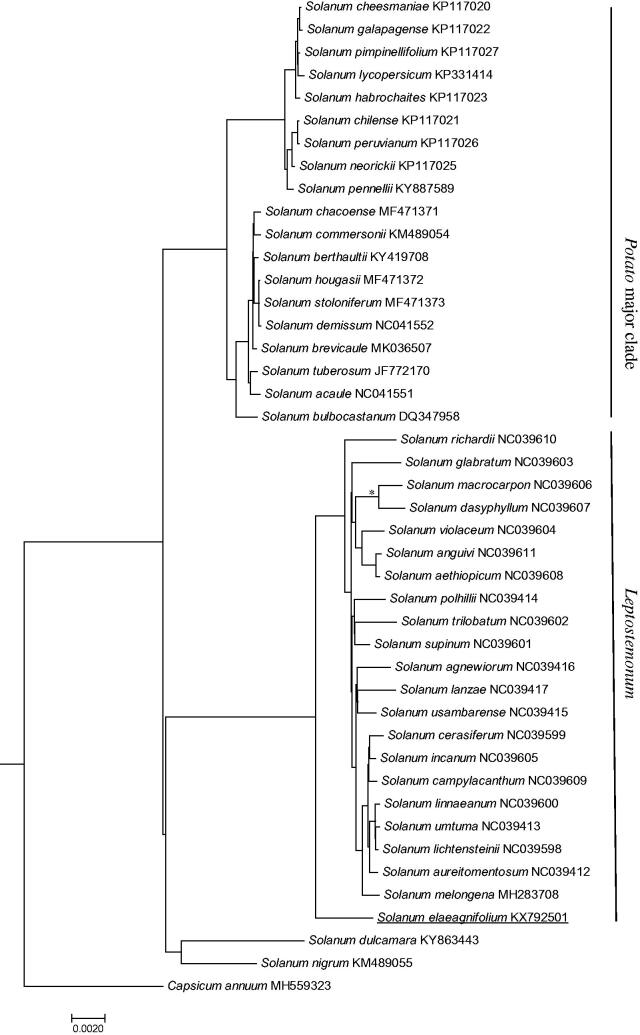
Phylogenetic relationships among *S. elaeagnifolium* (underlined) and 42 other *Solanum* species, inferred from the complete chloroplast. * Branches with Bayesian posterior probability lower than 90%.

## Data Availability

The data that support the findings of this study are openly available in GenBank at http://www.ncbi.nlm.nih.gov, accession number KX792501.

## References

[CIT0001] Doyle JJ. 1987. A rapid DNA isolation procedure for small quantities of fresh leaf tissue. Phytochem Bull. 19:11–15.

[CIT0002] Gopurenko D, Wang A, Zhu X, Lepschi BJ, Wu H. 2014. Origins and diversity of exotic silverleaf nightshade (Solanum elaeagnifolium) present in Australia as determined by sequence analysis of a chloroplast intergenic spacer region. In: Baker M, editor. 19th Australasian Weeds Conference. Hobart, TAS, Australia: Tasmanian Weed Society. p. 392–395.

[CIT0003] Hosking JR, Sainty GR, Jacobs SWL. 1996. Certainty and uncertainty in plant identification. In: Shepherd RCH, editor. 11th Australian Weeds Conference. Melbourne, VIC, Australia: Weed Science Society of Victoria. p. 464–467.

[CIT0004] Katoh K, Standley DM. 2013. MAFFT multiple sequence alignment software version 7: improvements in performance and usability. Mol Biol Evol. 30(4):772–780. 23329690 10.1093/molbev/mst010 PMC3603318

[CIT0005] Liu C, Shi L, Zhu Y, Chen H, Zhang J, Lin X, Guan X. 2012. CpGAVAS, an integrated web server for the annotation, visualization, analysis, and GenBank submission of completely sequenced chloroplast genome sequences. BMC Genom. 13:715. 10.1186/1471-2164-13-715 PMC3543216 23256920

[CIT0006] Luo R, Liu B, Xie Y, Li Z, Huang W, Yuan J, He G, Chen Y, Pan Q, Liu Y, et al. 2012. SOAPdenovo2: an empirically improved memory-efficient short-read de novo assembler. Gigascience. 1(1):18. 23587118 10.1186/2047-217X-1-18 PMC3626529

[CIT0007] Raubeson LA, Jansen RK. 2005. Chloroplast genomes of plants. In: Henry RJ, editor. Plant diversity and evolution: genotypic and phenotypic variation in higher plants. Wallingford: CABI Publishing. p. 45–68.

[CIT0008] Ronquist F, Huelsenbeck J. 2003. MrBayes 3: Bayesian phylogenetic inference under mixed models. Bioinformatics. 19(12):1572–1574. 12912839 10.1093/bioinformatics/btg180

[CIT0009] Stanton R, Heap JW, Carter RJ, Wu H. 2009. *Solanum elaeagnifolium*. In: Panetta FD, editor. The biology of Australian weeds. Melbourne: RG and FJ Richardson. p. 274–293.

[CIT0010] Zhu X, Gopurenko D, Haegi LAR, Wu H. 2018. Molecular identification of *Solanum elaeagnifolium* in Australia using DNA barcoding, a solution for better management. In: 21st Australasian Weeds Conference. Sydney, New South Wales: Weed Society of New South Wales. p. 307–311.

[CIT0011] Zhu XC, Burrows G, Wu H, Raman H, Stanton R, Lemerle D. 2011. Identification of silverleaf nightshade using microsatellite markers and microstructure. In: Adkins S, McFadden R, editors. 23rd Asian-Pacific Weed Science Society Conference. Cairns, QLD, Australia. Weed Society of Queensland. p. 604–609.

